# Supplementary material for: A bioactivated in vivo assembly nanotechnology fabricated NIR probe for small pancreatic tumor intraoperative imaging
Source: Nat Commun. 2022 Jan 20;13:418. doi: 10.1038/s41467-021-27932-y (PMC8776730; doi:10.1038/s41467-021-27932-y)
Supplement: Supplementary file 1 — Supplementary Information [file 41467_2021_27932_MOESM1_ESM.pdf]

# Supplementary Materials for

## A bioactivated *in vivo* assembly nanotechnology fabricated NIR probe for small pancreatic tumor intraoperative imaging

Han Ren[1‡], Xiang-Zhong Zeng[1,2,3‡], Xiao-Xiao Zhao[1‡], Da-yong Hou[1,4,5], Haodong Yao[6], Muhammad Yaseen[7], Lina Zhao[6], Wan-hai Xu[4,5], Hao Wang[1], and Li-Li Li[1]*

[1] CAS Center for Excellence in Nanoscience, CAS Key Laboratory for Biological Effects of Nanomaterials and Nanosafety, National Center for Nanoscience and Technology (NCNST), 100190 Beijing, China.

[2] Center of Materials Science and Optoelectronics Engineering, University of Chinese Academy of Sciences (UCAS), 100049 Beijing, China.

[3] Academy for Advanced Interdisciplinary Studies, Peking University, 100871 Beijing, China.

[4] Department of Urology, The Fourth Hospital of Harbin Medical University, Heilongjiang Key Laboratory of Scientific Research in Urology, 150001 Harbin, China.

[5] NHC Key Laboratory of Molecular Probes and Targeted Diagnosis and Therapy, Harbin Medical University, Harbin, 150001, China.

[6] Institute of High Energy Physics, Chinese Academy of Sciences (CAS), 100049 Beijing, China.

[7] Institute of Chemical Sciences, University of Peshawar, 25120, KP, Pakistan.

[‡] H. Ren, X.-Z. Zeng, and X.-X. Zhao contributed equally to this work.

*Corresponding author. Email: lill@nanoctr.cn

**This PDF file includes:**

Supplementary Table 1 and Figs. 1-28

# Content

**Supplementary Table 1** | Secondary structure proportions of **M1**, **M3** and **R-M1** calculated by Reed's Reference based on CD spectra........1

**Supplementary Fig. 1** | Chemical structure and synthesis of **M1:** mPEG-GPAKLVFFGC(IR783)GRGD:........2

**Supplementary Fig. 2** | Chemical structure and mass spectra of **M1**: mPEG-GPAKLVFFGC($IR_{783}$)GRGD........3

**Supplementary Fig. 3** | Chemical structure and mass spectra of **M2**: mPEG-AGGKLVFFGC($IR_{783}$)GRGD........4

**Supplementary Fig. 4** | **Chemical** structure and mass spectra of **M3**: mPEG-GPAKLVFFGCGRGD........5

**Supplementary Fig. 5** | **Chemical** structure and mass spectra of **R-M1**: AKLVFFGC($IR_{783}$)GRGD........6

**Supplementary Fig. 6** | **Chemical** structure and mass spectra of **M4**: mPEG-GPAKLVFFGC($IR_{783}$)GDTG........7

**Supplementary Fig. 7** | Chemical structure and mass spectra of **M5**: mPEG-AGGKLVFFGCGRGD........8

**Supplementary Fig. 8** | Chemical structure and mass spectra of **R-M3**: AKLVFFGCGRGD........9

**Supplementary Fig. 9** | The critical aggregation concentration (CAC) of **M3** and **M5**........10

**Supplementary Fig. 10** | The critical aggregation concentration (CAC) of **R-M3** and **R-M1**........11

**Supplementary Fig. 11** | The radius of gyration (RGYR) (a) and root mean square fluctuation (RMSF) (b) of AKLVFFGCGRGD sequences of **M1** and **R-M1**........12

**Supplementary Fig. 12** | The ANS staining dynamic fibril growth curve of **R-M1**........13

**Supplementary Fig. 13** | The TEM image of self-assembled β-sheet nanofibers of **M1** (100 μM) after tailoring by FAP-α in buffer for 12 h........14

**Supplementary Fig. 14** | HPLC curve of **M3** co-incubated with inactivated FAP-α for 12h........15

**Supplementary Fig. 15** | The TEM image of **M1** co-incubated with Miapaca-2 cell lysis for 12 h........16

**Supplementary Fig. 16** | TEM image of **M1** co-incubated with inactivated FAP-α for 12h........17

**Supplementary Fig. 17** | MALDI-TOF mass spectra of **M1** co-incubated with Miapaca-2 cell lysis for 2 h........18

**Supplementary Fig. 18** | Representative confocal images in three independ experiment of **M1** with BIVA effect on Miapaca-2 cells after incubation for 2 h........19

**Supplementary Fig. 19** | CLSM images of Miapaca-2 cells treated under different conditions........21

**Supplementary Fig. 20** | The quantitative results of the migrated cells after treatment of PBS (blank), **M2**, **M3** and **M1**............................................................................................................................................. 22

**Supplementary Fig. 21** | Cell viability assay of Miapaca-2 cells treated with a series concentration of **M1** and **M2** for 24 h............................................................................................................................................23

**Supplementary Fig. 22** | 3D reconstruction PA images of tumor-bearing mice injected with **M1** (16 mg/kg) through tail vein...........................................................................................................................................24

**Supplementary Fig. 23** | Images of **M1** and **M2** molecules *in situ* in nude mice with pancreatic cancer were measured at 12 h post injection.............................................................................................................................25

**Supplementary Fig. 24** | The *in vivo* and *in situ* NIR images of orthotopic pancreatic tumors by **M1** and **M2**,........... 26

**Supplementary Fig. 25** | The 3D quantitative fluorescence intensity distribution of small size *ex vivo* orthotopic pancreatic tumor images of **M1**.............................................................................................................27

**Supplementary Fig. 26** | The congo red stained of tumor histologic section in mice treated with **M1** and **M2**...........28

**Supplementary Fig. 27** | Fluorescence images of tumor histologic section treated with **M1** (16 mg/kg) for 48 h........29

**Supplementary Fig. 28** | The congo red tumor stained histologic section in mice treated with **M1** (16 mg/kg) for 48 h ...........................................................................................................................................30

**Supplementary Table 1** | Secondary structure proportions of **M1**, **M3** and **R-M1** calculated by Reed's Reference based on CD spectra.

| | M1 | M3 | R-M1 |
|---|---|---|---|
| Helix | 0 | 45 | 0.5 |
| Beta | 32.2 | 38.7 | 72 |
| Turn | 1.7 | 0 | 27.5 |
| Random | 66.1 | 16.3 | 0 |
| RMS | 14.093 | 5.66 | 23.908 |

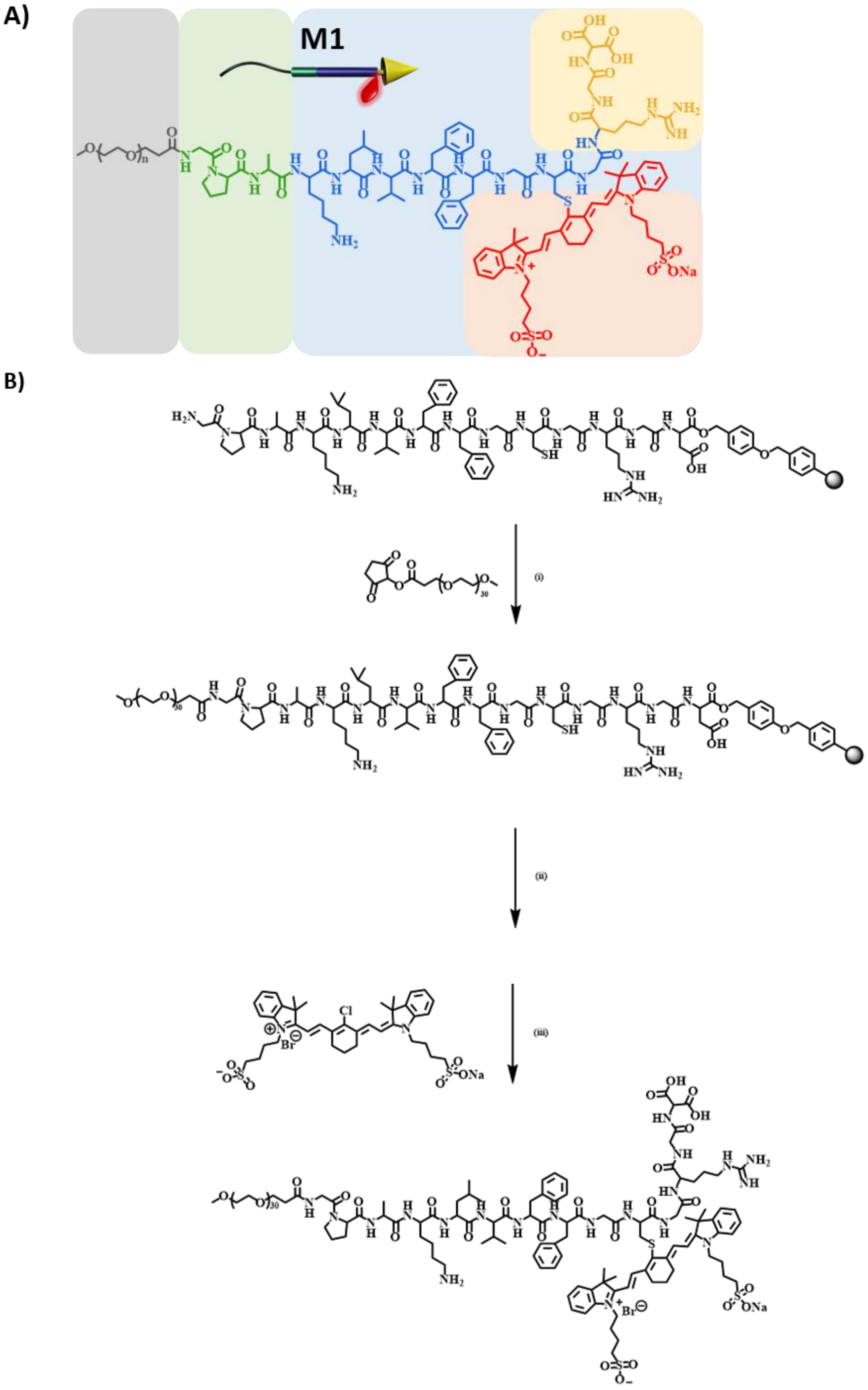

**Supplementary Fig. 1** │ A) Chemical structure of **M1**: mPEG-GPAKLVFFGC(IR783)GRGD. B) Synthesis of **M1**: (i). 5% NMM/DMF, 10equiv. HBTU, pH9.0, 24h. (ii). TFA/TIPS/1,2-ethanedithiol/Diwater (92.5%/2.5%/2.5%/2.5%, v/v), stirring, ice bath, 3h. (iii). Tris buffer, pH8.5, 12h.

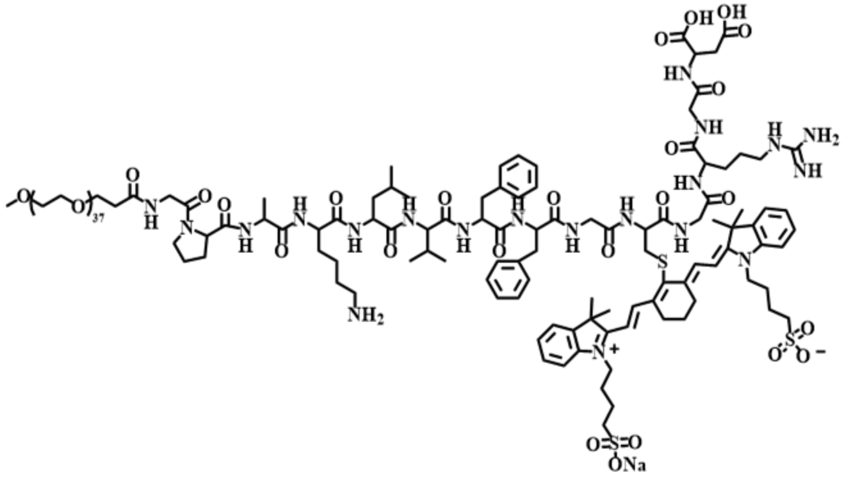

**M1** : mPEG-GPAKLVFFGC(IR783)GRGD

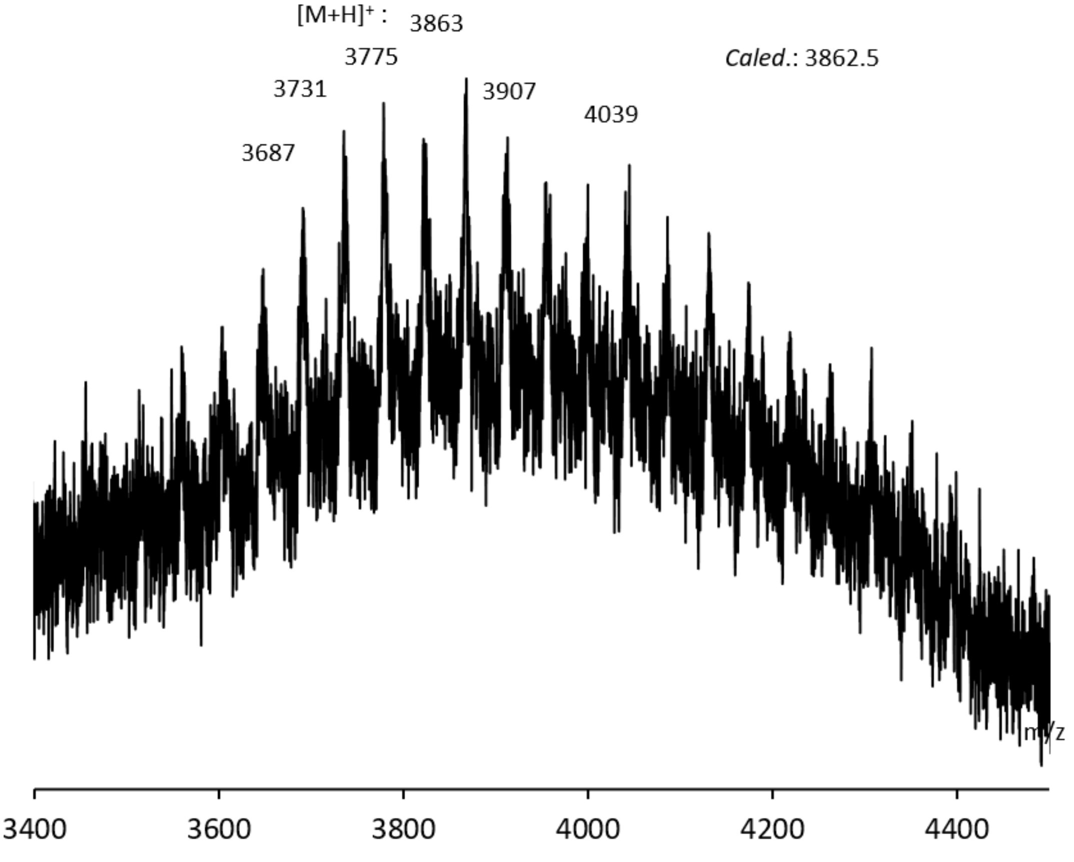


**Supplementary Fig. 2** | Chemical structure and MALDI-TOF mass spectra of **M1**: mPEG-GPAKLVFFGC($IR_{783}$)GRGD.

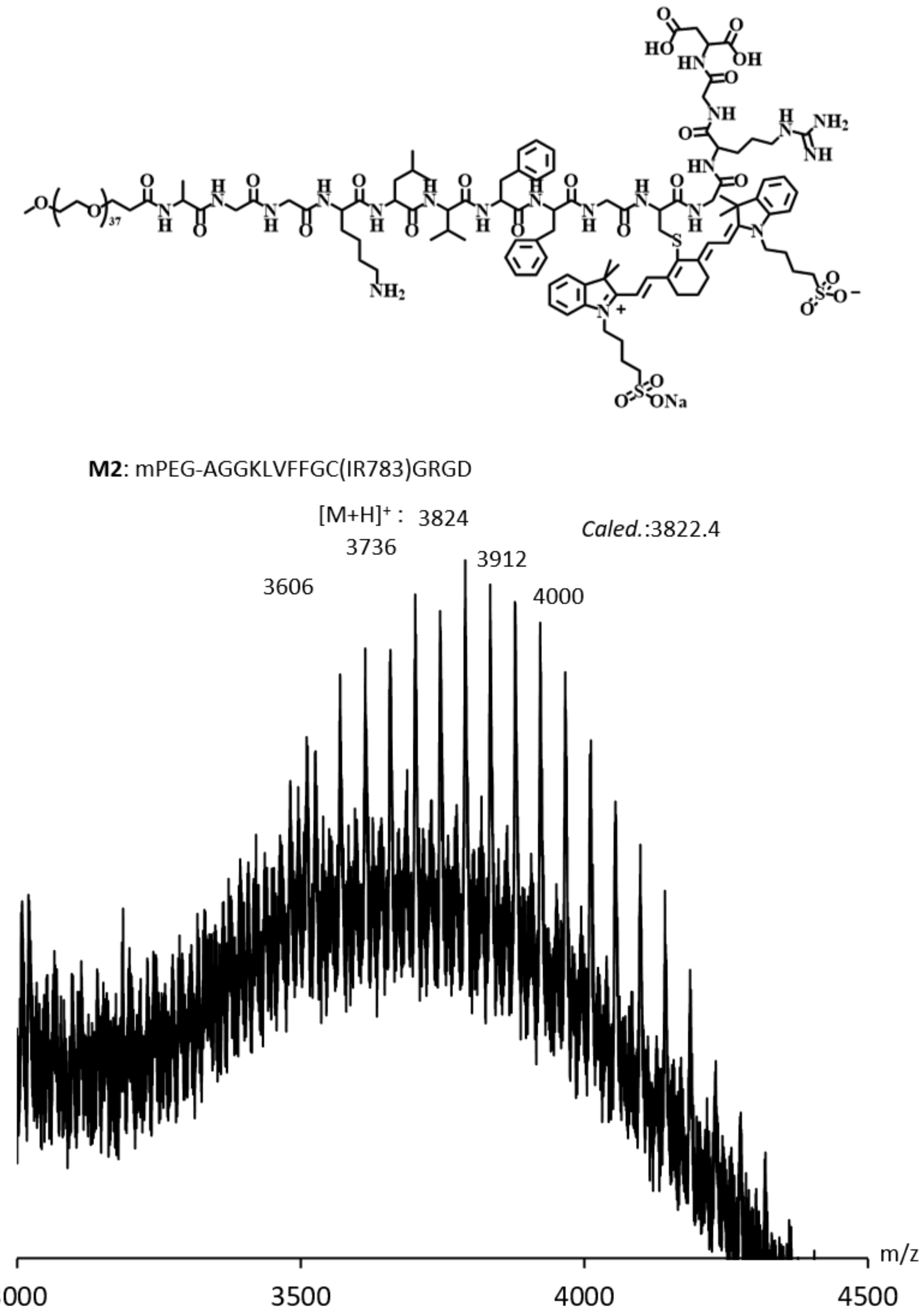


**Supplementary Fig. 3** | Chemical structure and MALDI-TOF mass spectra of **M2**: mPEG-AGGKLVFFGC($IR_{783}$)GRGD.

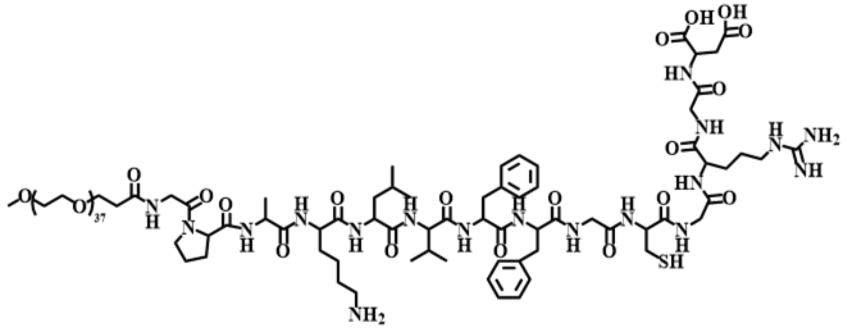


**M3**: mPEG-GPAKLVFFGCGRGD

*Caled.*: 3148.5

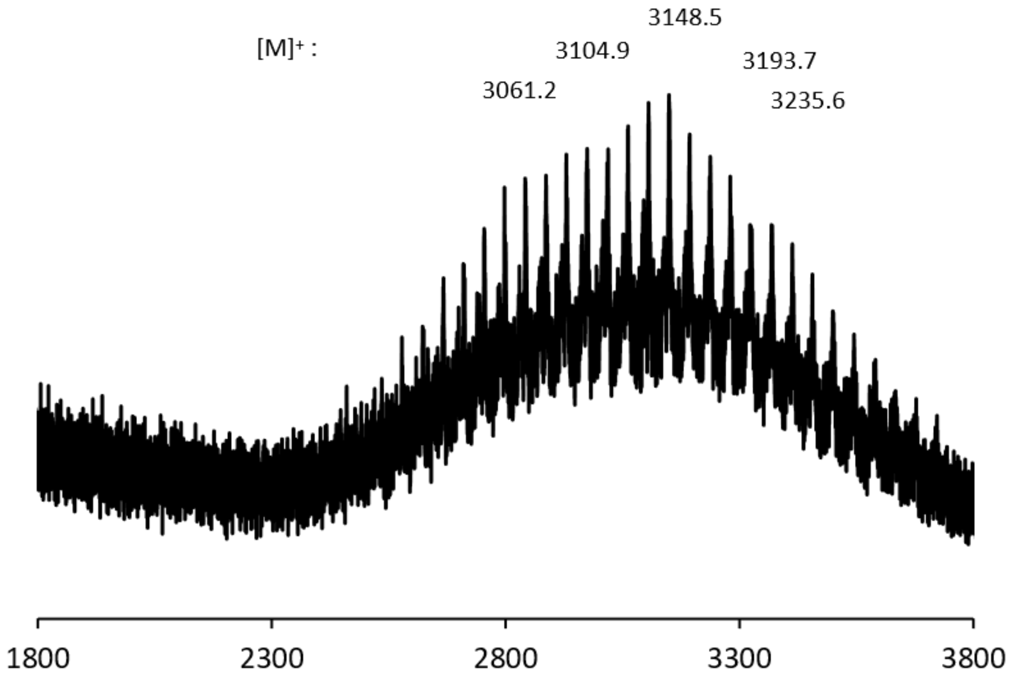


**Supplementary Fig. 4** | **Chemical** structure and MALDI-TOF mass spectra of **M3**: mPEG-GPAKLVFFGCGRGD.

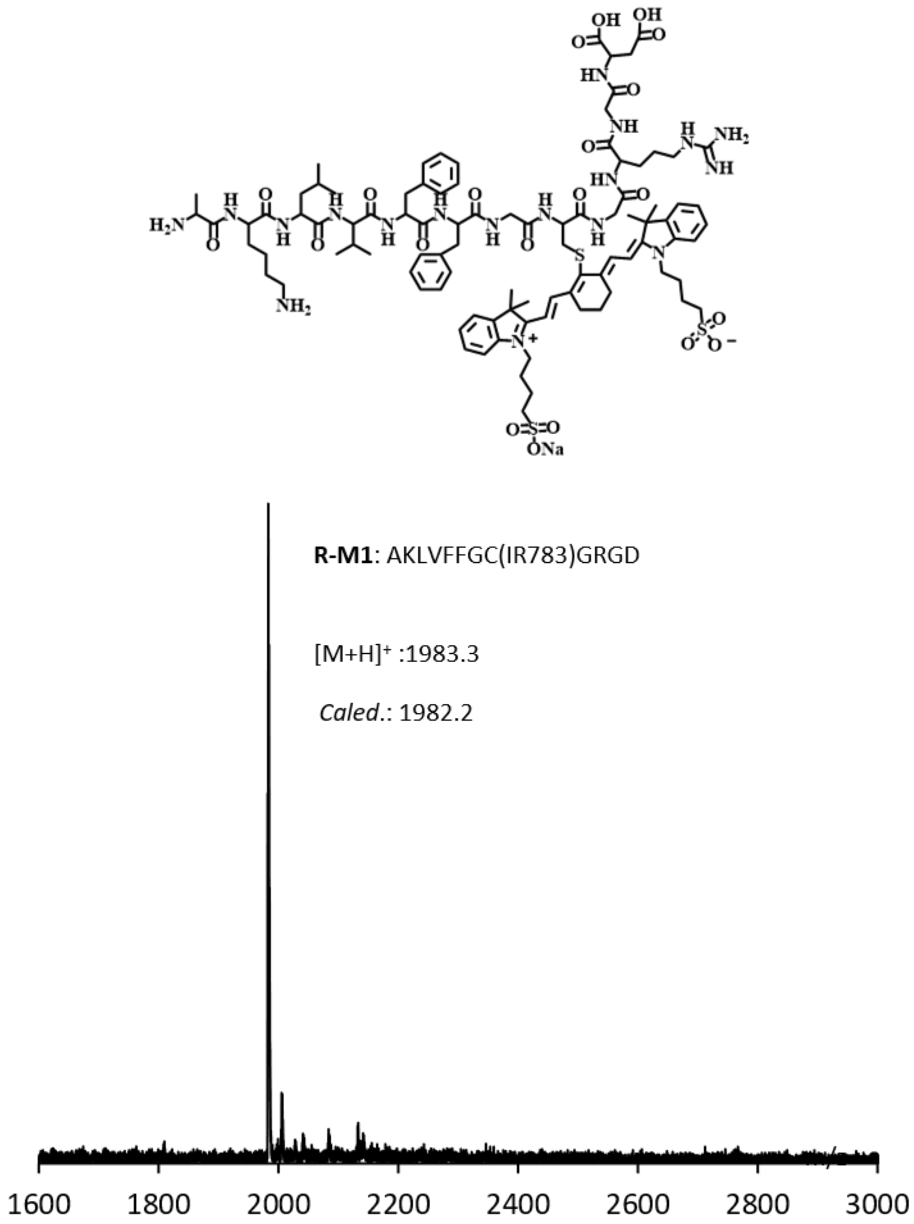


**Supplementary Fig. 5 | Chemical** structure and MALDI-TOF mass spectra of **R-M1**: AKLVFFGC($IR_{783}$)GRGD.

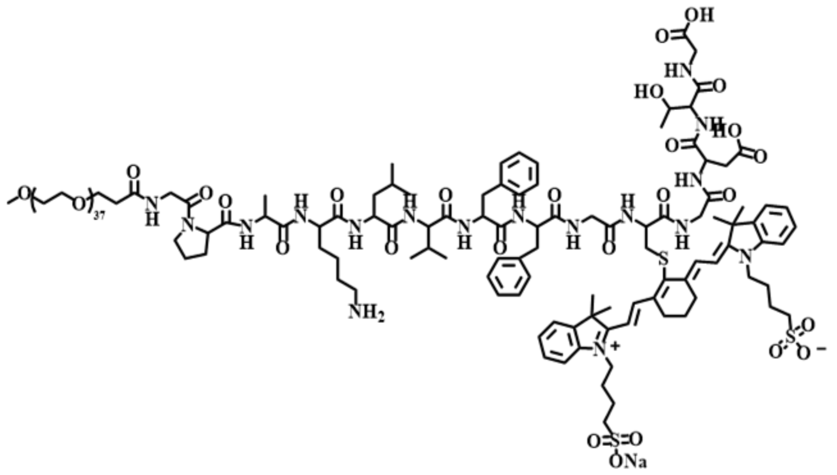


**M4**: mPEG-GPAKLVFFGC(IR783)GDTG

*Caled*.: 3823.1

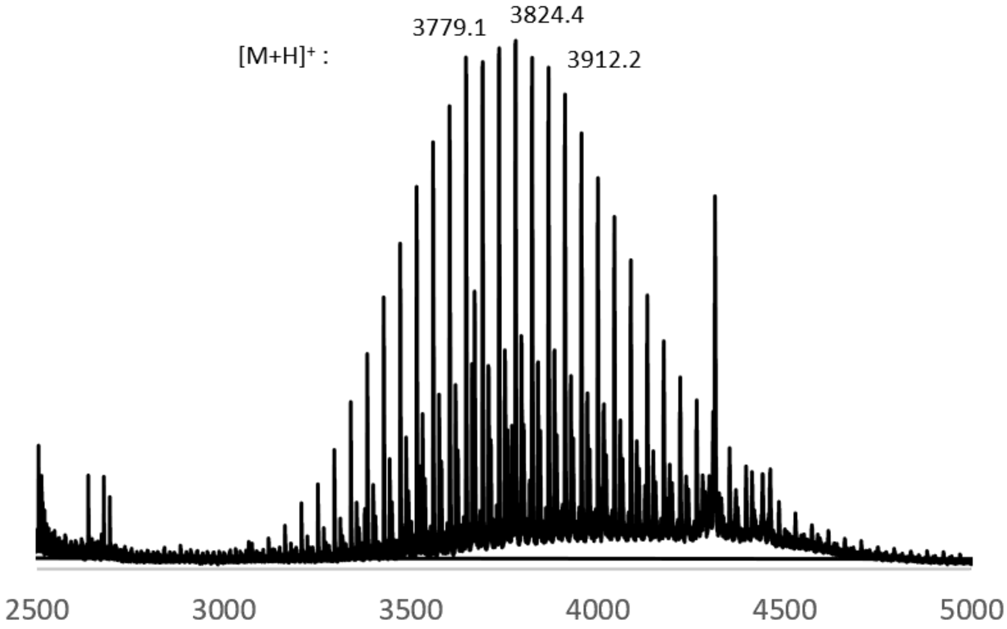


**Supplementary Fig. 6** | Chemical structure and MALDI-TOF mass spectra of **M4**: mPEG-GPAKLVFFGC($IR_{783}$)GDTG.

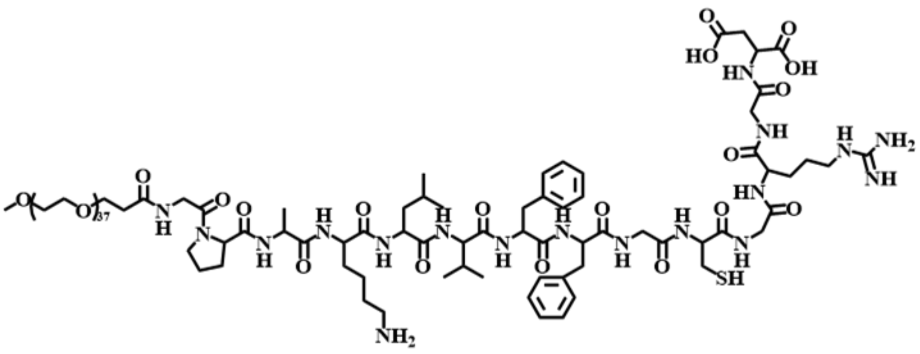

**M5:** : mPEG-AGGKLVFFGCGRGD

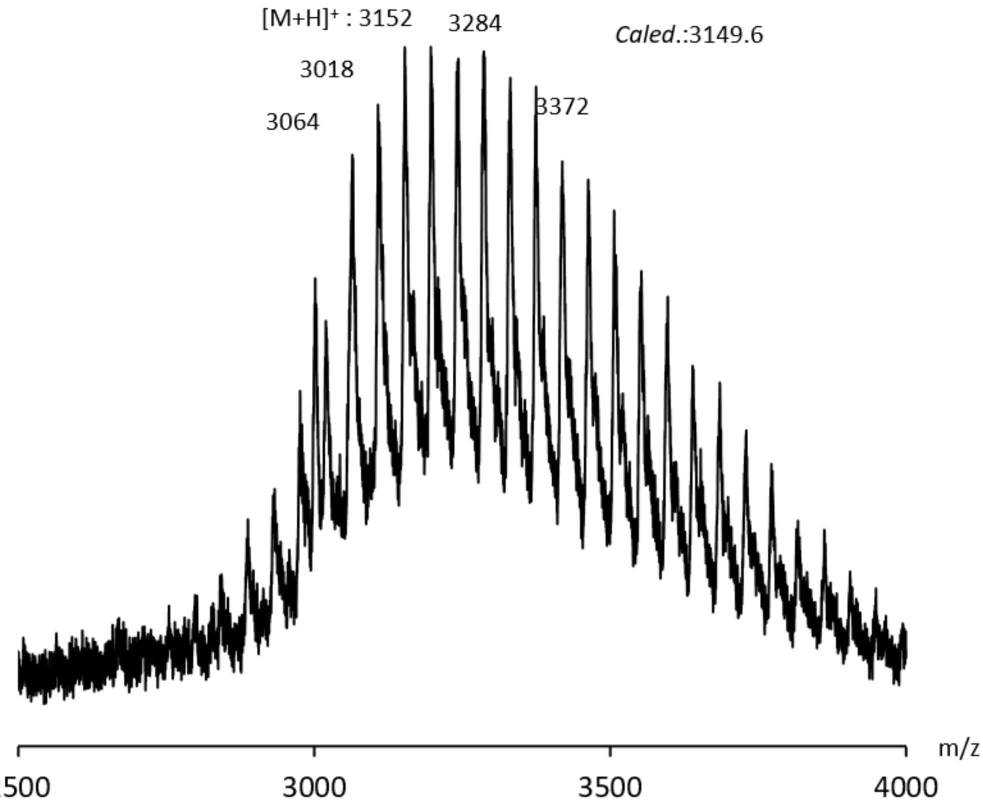


**Supplementary Fig. 7** | Chemical structure and MALDI-TOF mass spectra of **M5**: mPEG-AGGKLVFFGCGRGD.

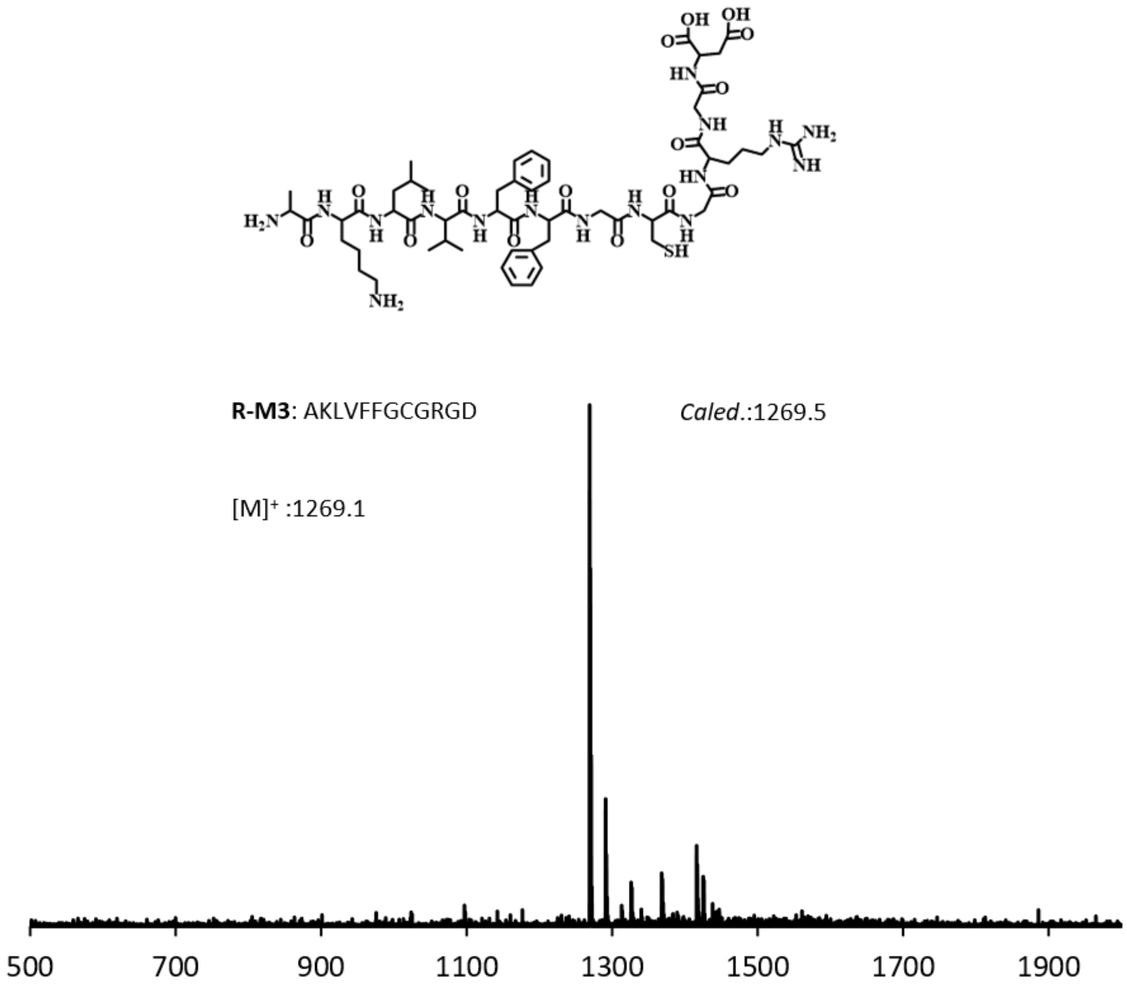


**Supplementary Fig. 8** | Chemical structure and MALDI-TOF mass spectra of **R-M3**: AKLVFFGCGRGD.

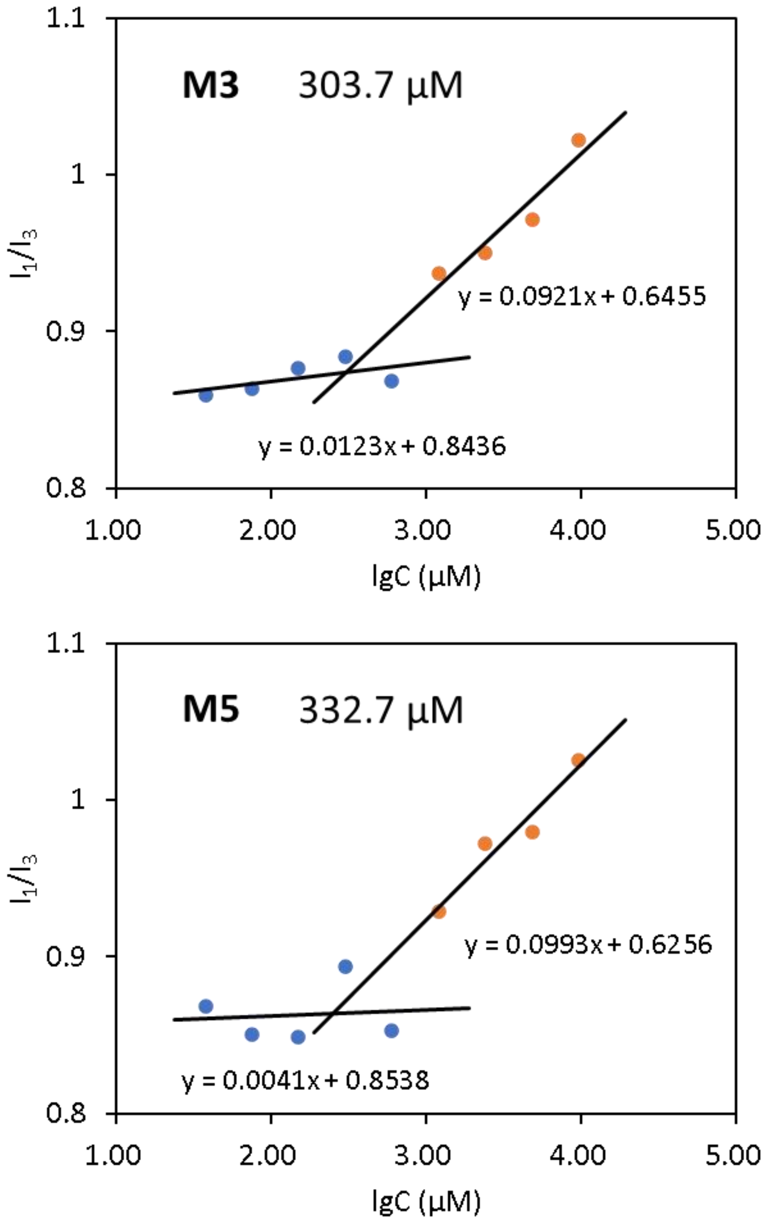


**Supplementary Fig. 9** | The critical aggregation concentration (CAC) of **M3** and **M5** was measured by using pyrene as a probe. The ratio of peak $I_1$ to peak $I_3$ can be obtained by measuring the fluorescence of pyrene in different concentrations of molecules. Experiments were repeated three times. The x axis is logarithm concentration and the y axis is the ratio of peak intensities of 374 nm and 383 nm. Ex= 334 nm.

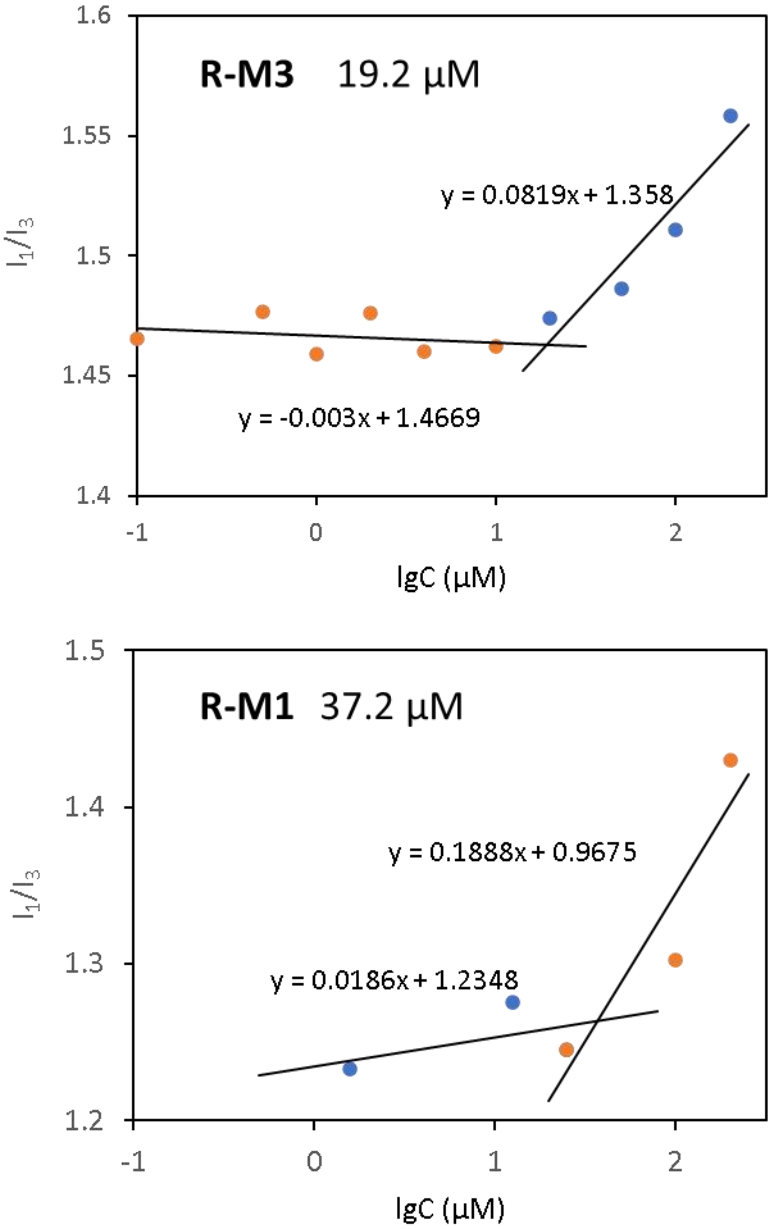


**Supplementary Fig. 10** | The critical aggregation concentration (CAC) of **R-M3** and **R-M1** was measured by using pyrene as a probe. The ratio of peak $I_1$ to peak $I_3$ can be obtained by measuring the fluorescence of pyrene in different concentrations of molecules. To figure out the CAC of the molecule. Experiments were repeated three times. The x axis is logarithm concentration and the y axis is the ratio of peak intensities of 374 nm and 383 nm. Ex= 334 nm.

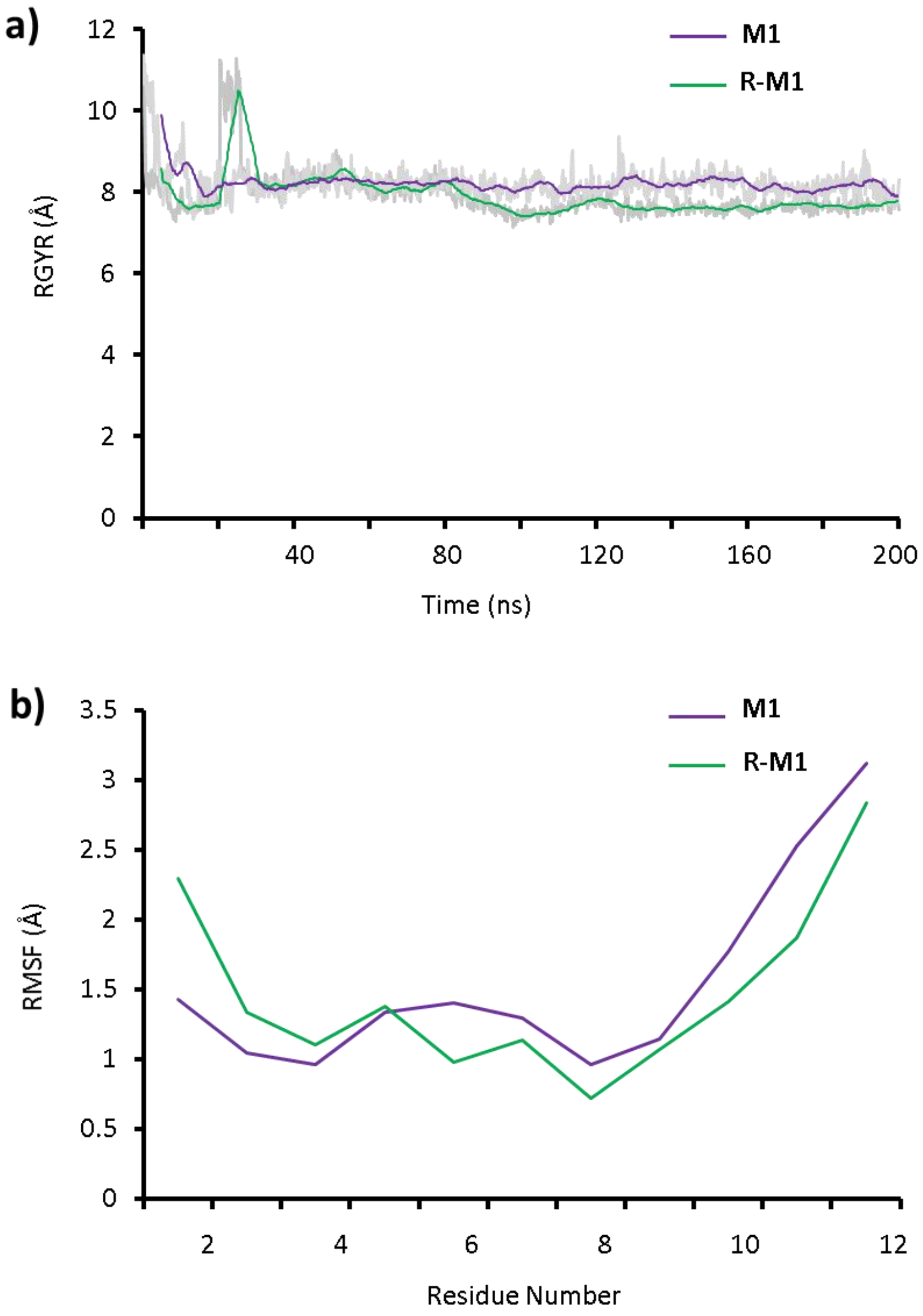


**Supplementary Fig. 11** | The radius of gyration (RGYR) (a) and root mean square fluctuation (RMSF) (b) of AKLVFFGCGRGD sequences of **M1** and **R-M1**.

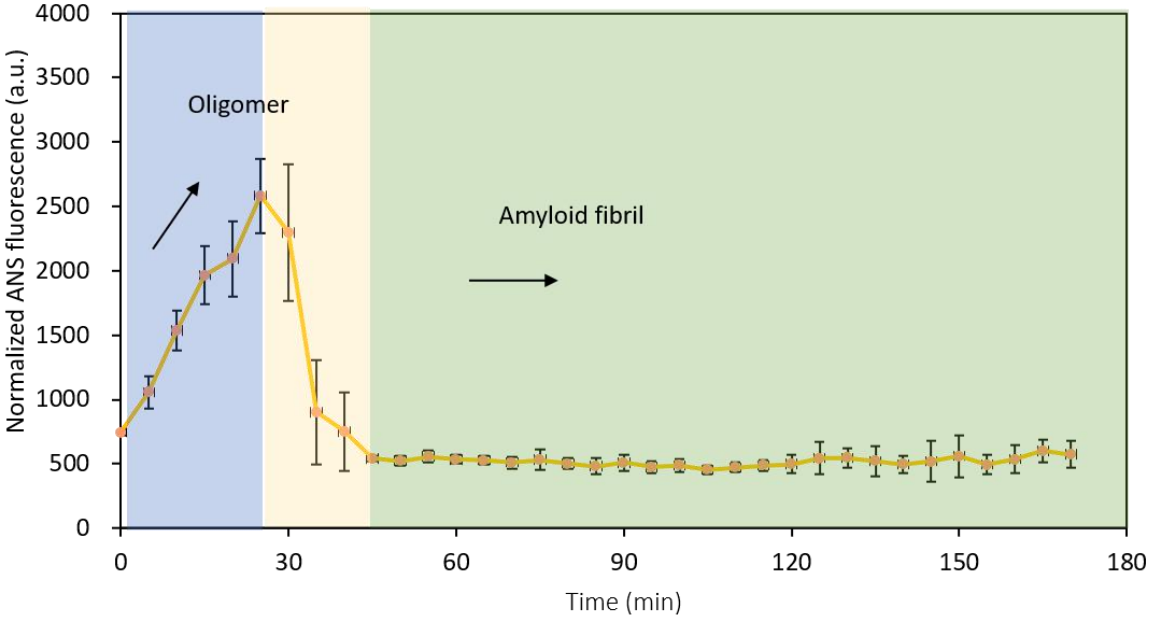


**Supplementary Fig. 12** | The ANS staining dynamic fibril growth curve of **R-M1**. ANS was added to an aqueous solution of **R-M1** (100 μM) and fluorescence was measured immediately. The same experiment was carried out four times. The mean of results of four parallel experiments is shown and data were expressed as mean ± SD (n=4).

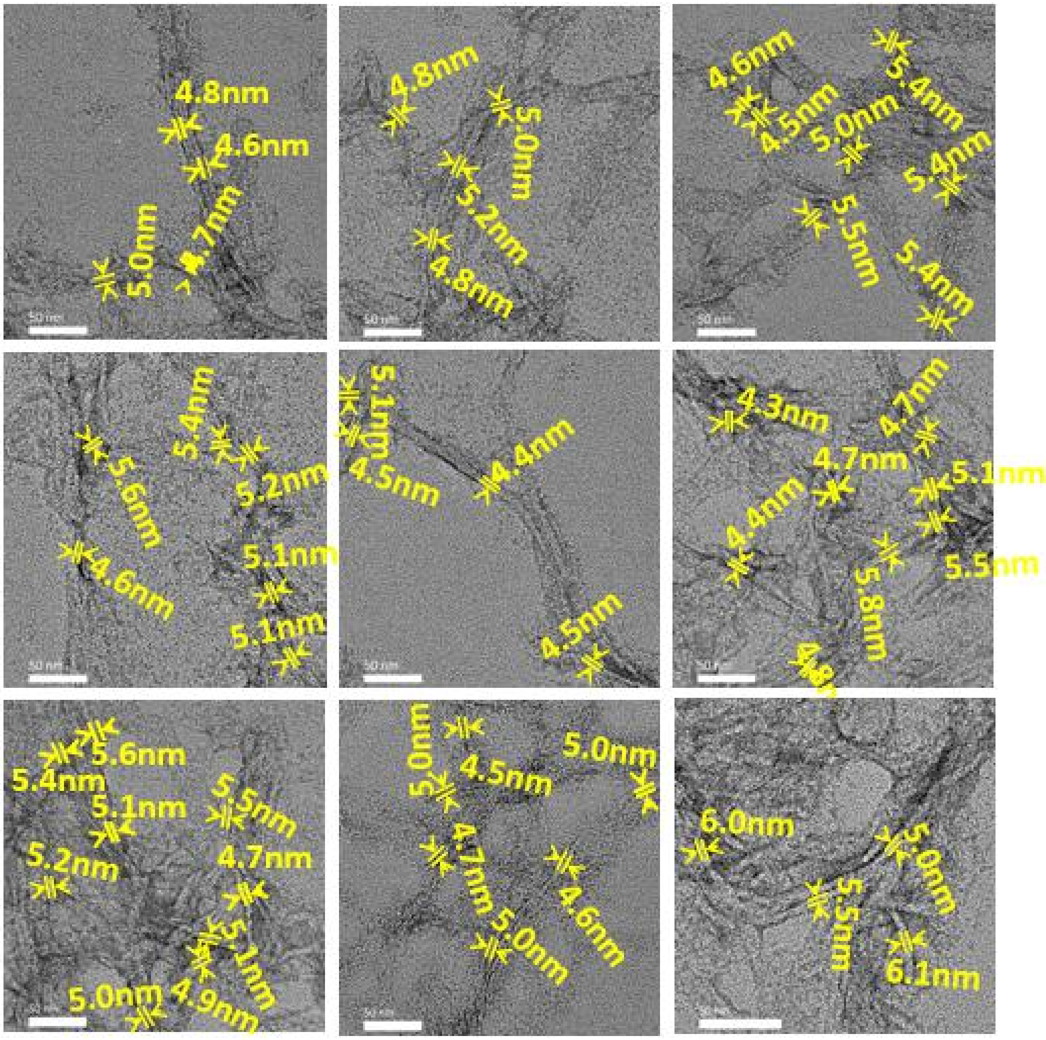


**Supplementary Fig. 13** | The TEM image of self-assembled β-sheet nanofibers of **M1** (100 μM) after tailoring by FAP-α (50 μM) in buffer for 12 h. Scale bar = 50 nm.

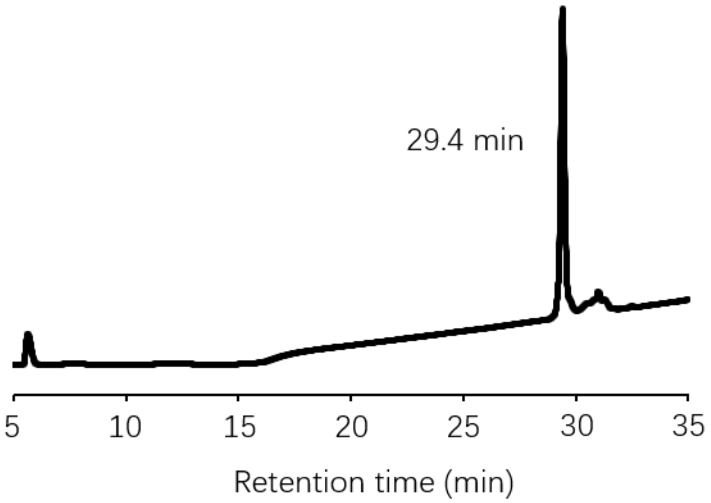


**Supplementary Fig. 14** | HPLC curve of **M3** co-incubated with inactivated FAP-α for 12h. The FAP-α was heat to 1 h to inactivate in advanced. HPLC: C18 column and a linear gradient of acetonitrile/water with 0.1% TFA from 10 %/ 90 % to 50 %/ 50 % and a flow speed of 1 mL/min in 35 min at 25 $^{o}$C.

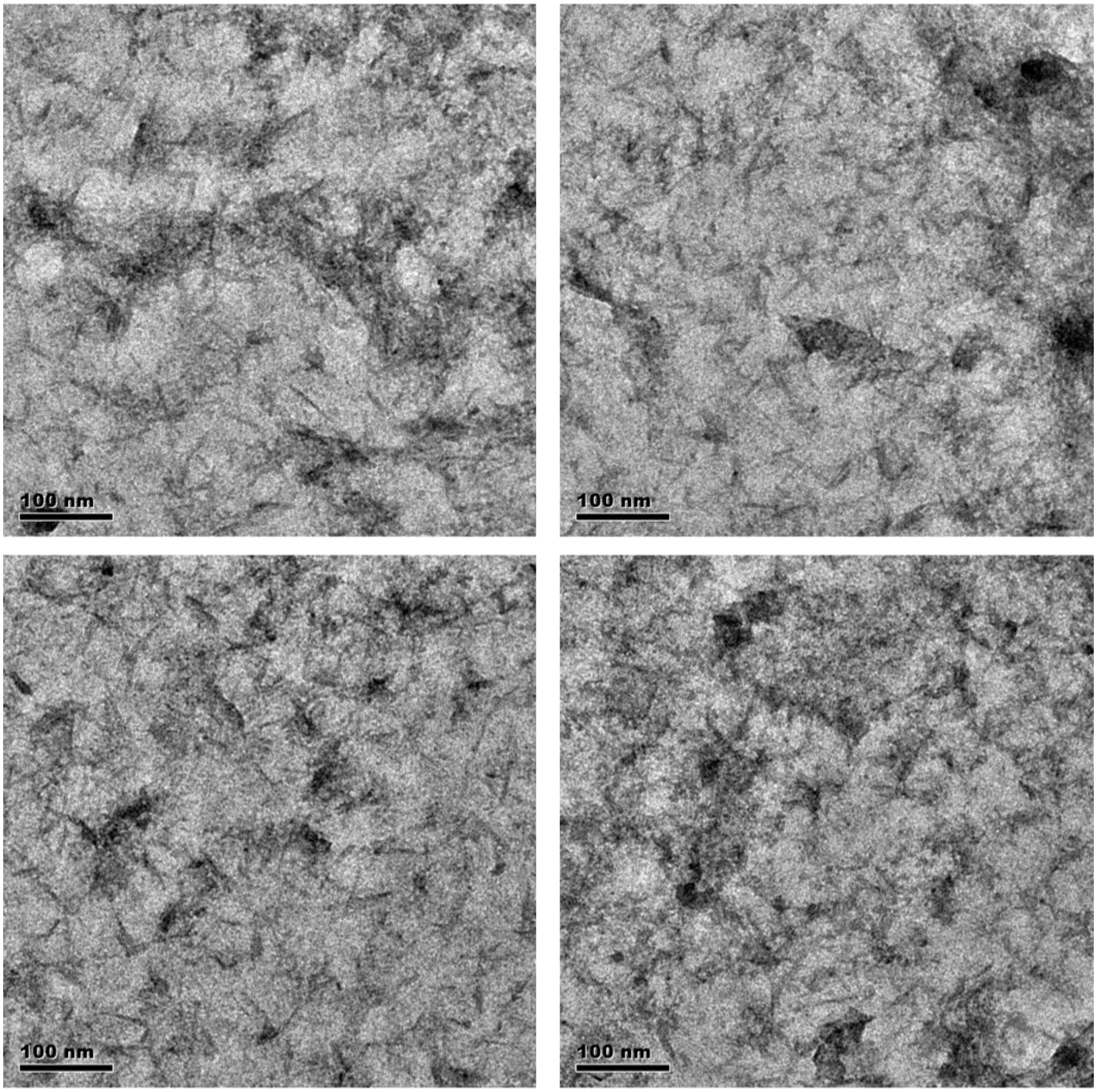


**Supplementary Fig. 15** | Representative TEM image of four independ experiment of **M1** (100 μM) co-incubated with Miapaca-2 cell lysis for 12 h. Scale bar: 100 nm.

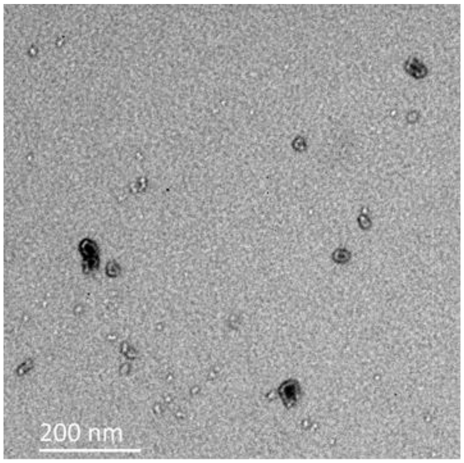


**Supplementary Fig. 16** | TEM image of **M1** (100 µM) co-incubated with inactivated FAP-α (50 µM) for 12h. The FAP-α was heat to 1 h to inactivate in advanced. Scale bar: 200 nm.

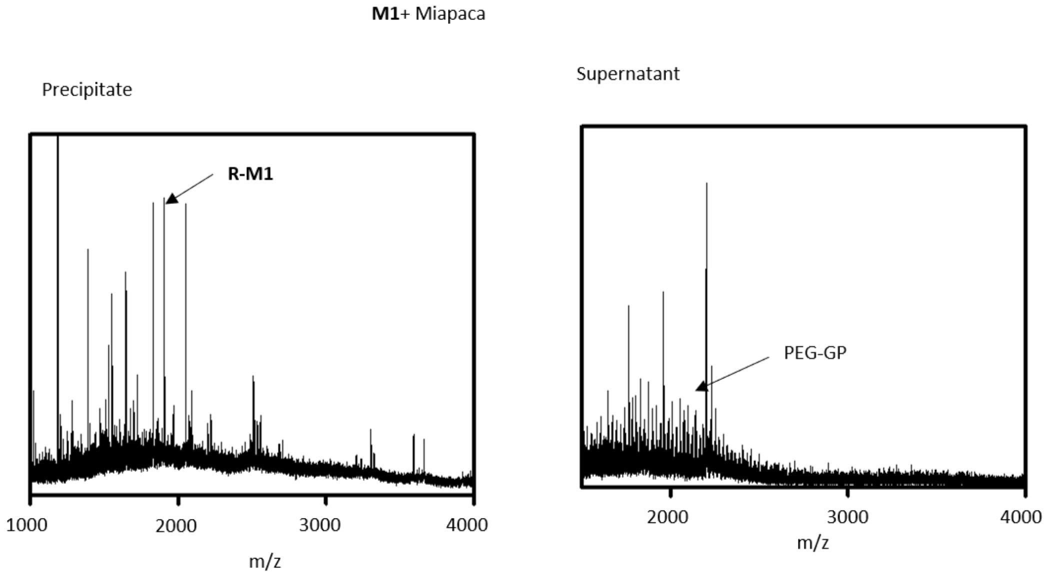


**Supplementary Fig. 17** | MALDI-TOF mass spectra of **M1** co-incubated with Miapaca-2 cell lysis for 2 h.

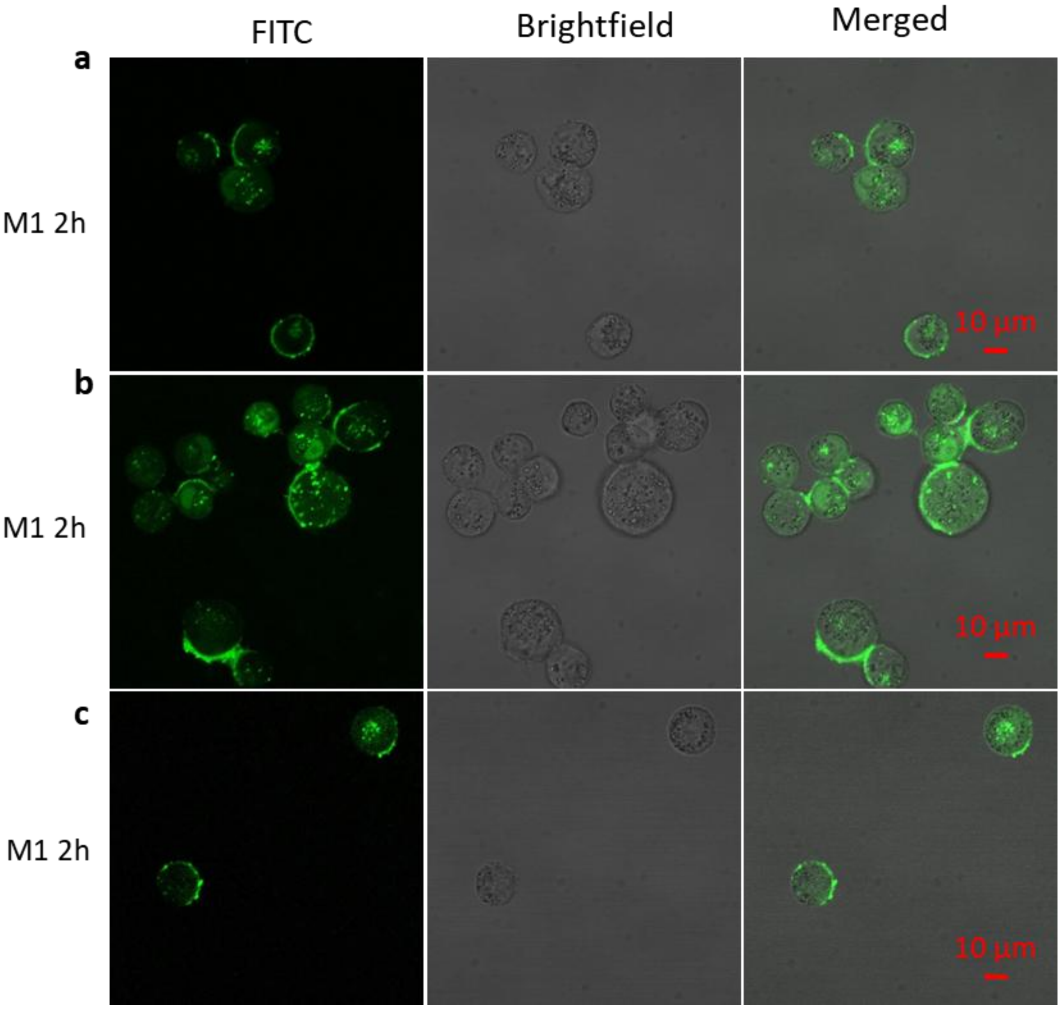


**Supplementary Fig. 18** | Representative confocal images in three independ experiment of **M1** (100 µM) with BIVA effect on Miapaca-2 cells after incubation for 2 h. Scale bar: 10 µm.

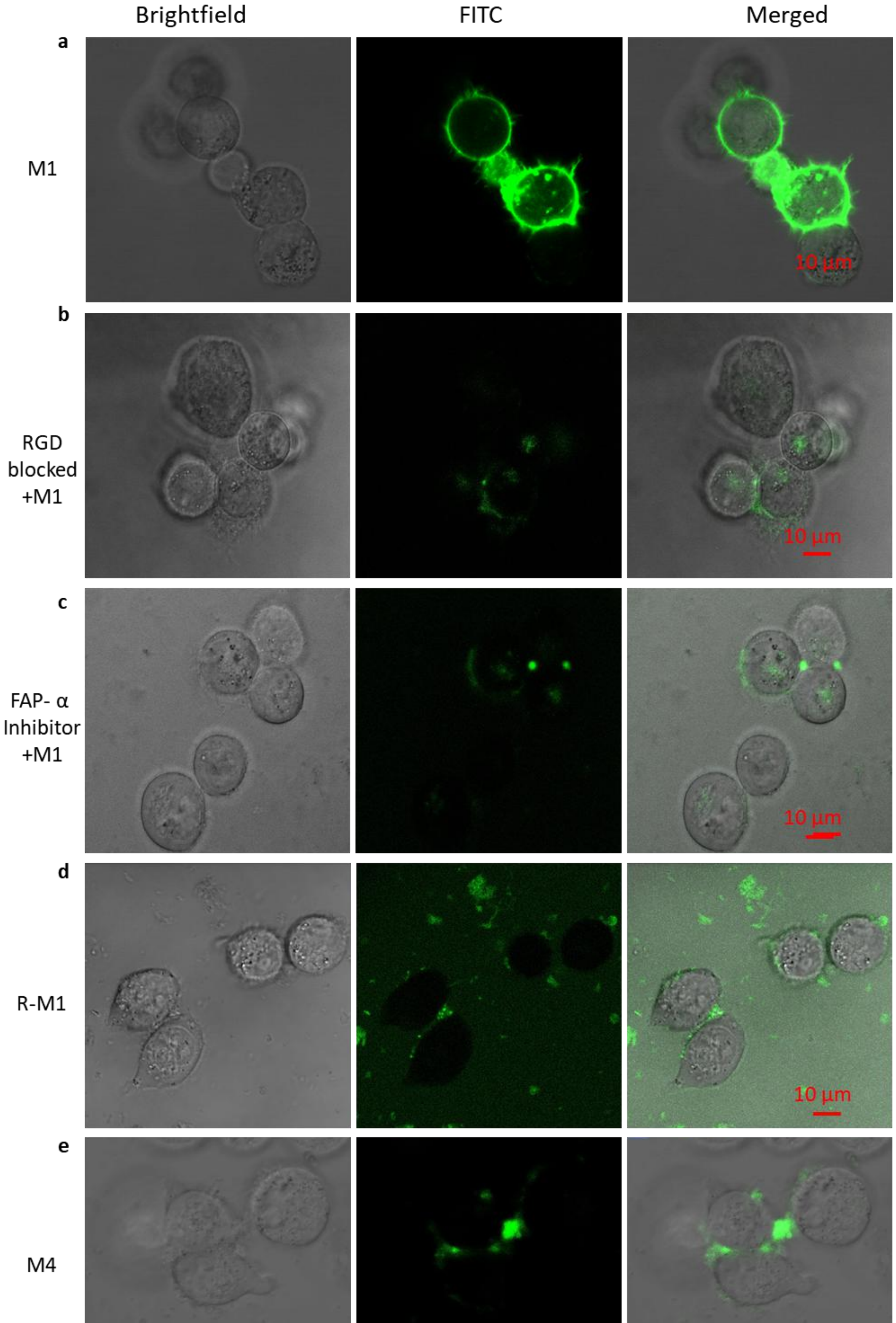
Brightfield
FITC
Merged
a
M1
10 µm
b
RGD
blocked
+M1
10 µm
c
FAP- α
Inhibitor
+M1
10 µm
d
R-M1
10 µm
e
M4

**Supplementary Fig. 19 |** CLSM images of Miapaca-2 cells treated with different molecules or **M1** under different conditions. All the molecules are labelled with FITC. **a,** CLSM images of **M1** with BIVA effect on Miapaca-2 cells after incubation for 1 h. **b, M1** incubated av integrins blocked Miapaca-2 cells for 1 h and washed by fresh DMEM for three times (Miapaca-2 cells ware treated by RGD for 1h at 50 μM and washed by fresh DMEM for three times before adding **M1**). **c, M1** incubated Fap-α inhibited Miapaca-2 cells for 1h and washed by fresh DMEM for three times (Miapaca-2 cells ware treated by Fap-α inhibitor Ac-Gly-BoroPro for 1h at 50 nM and wahed by fresh DMEM for three times before adding **M1**). d, **R-M1** incubated Miapaca-2 cells after incubation for 1 h without washing. **e, M4** incubated Miapaca-2 cells after incubation for 1 h and washed by fresh DMEM for three times. Green: FITC labelled molecules. Scale bar: 10 μm.

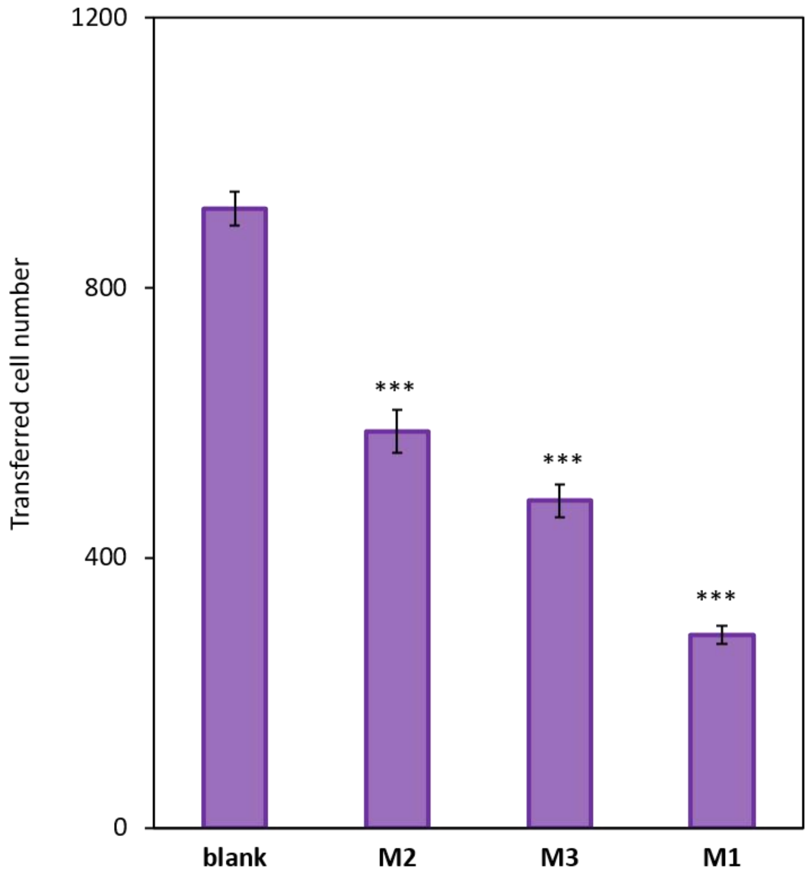


**Supplementary Fig. 20** | The quantitative results of the migrated cells after treatment of PBS (blank), **M2**, **M3** and **M1** (Fig 5g). The mean of results of five parallel experiments is shown and data were expressed as mean ± SD (n=5). $p_{M1}$ = 5.85E-06, $p_{M2}$ = 5.94E-05, $p_{M4}$ = 3.16 E-04, Statistical analysis: one-way ANOVA followed by post hoc Tukey's test, ***$p$<0.001.

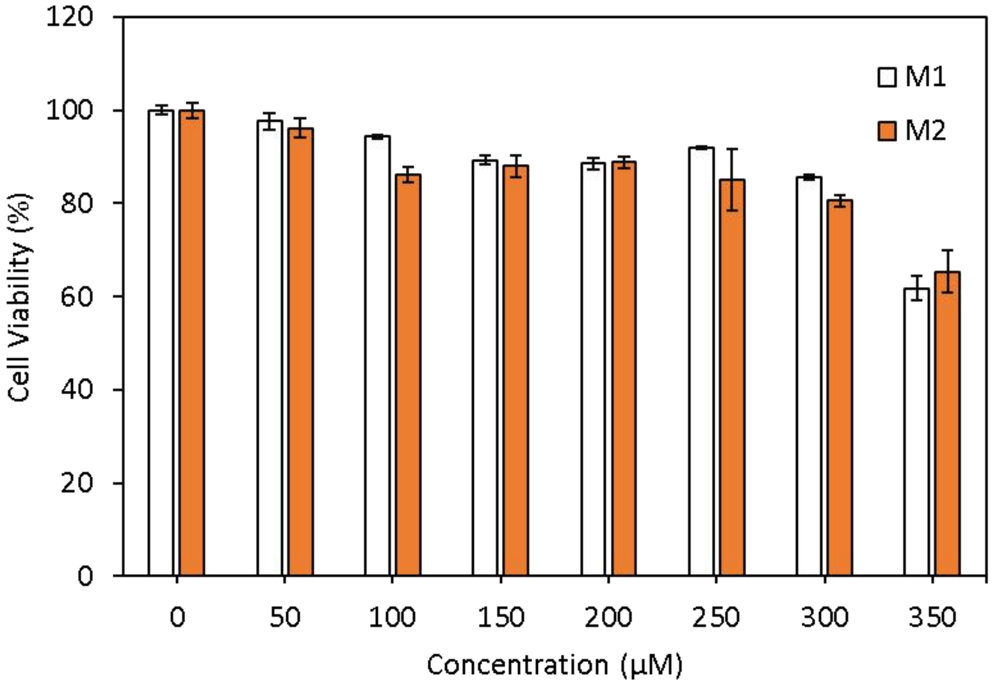


**Supplementary Fig. 21** | Cell viability assay of Miapaca-2 cells treated with a series concentration of **M1** and **M2** for 24 h. Tested by Cell Counting Kit-8 (Beyotime, C0039) The mean of results of six parallel experiments is shown and data were expressed as mean ± SD (n=6).

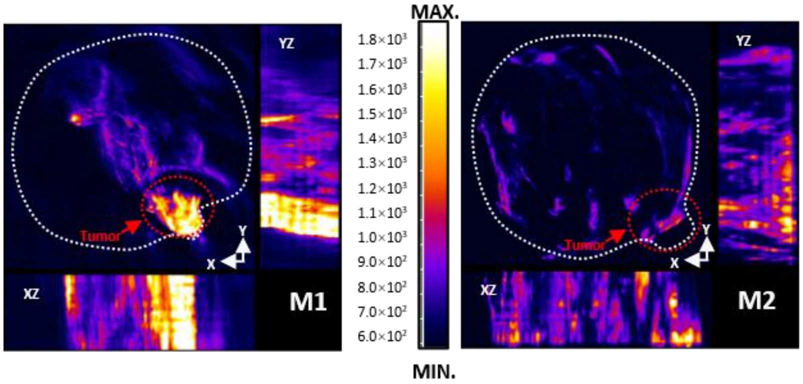


**Supplementary Fig. 22** | 3D reconstruction PA images of tumor-bearing mice injected with **M1** (16 mg/kg) through tail vein. PA images of mice were acquired with MOST (mode: MOST 128) at 12 h post injection.

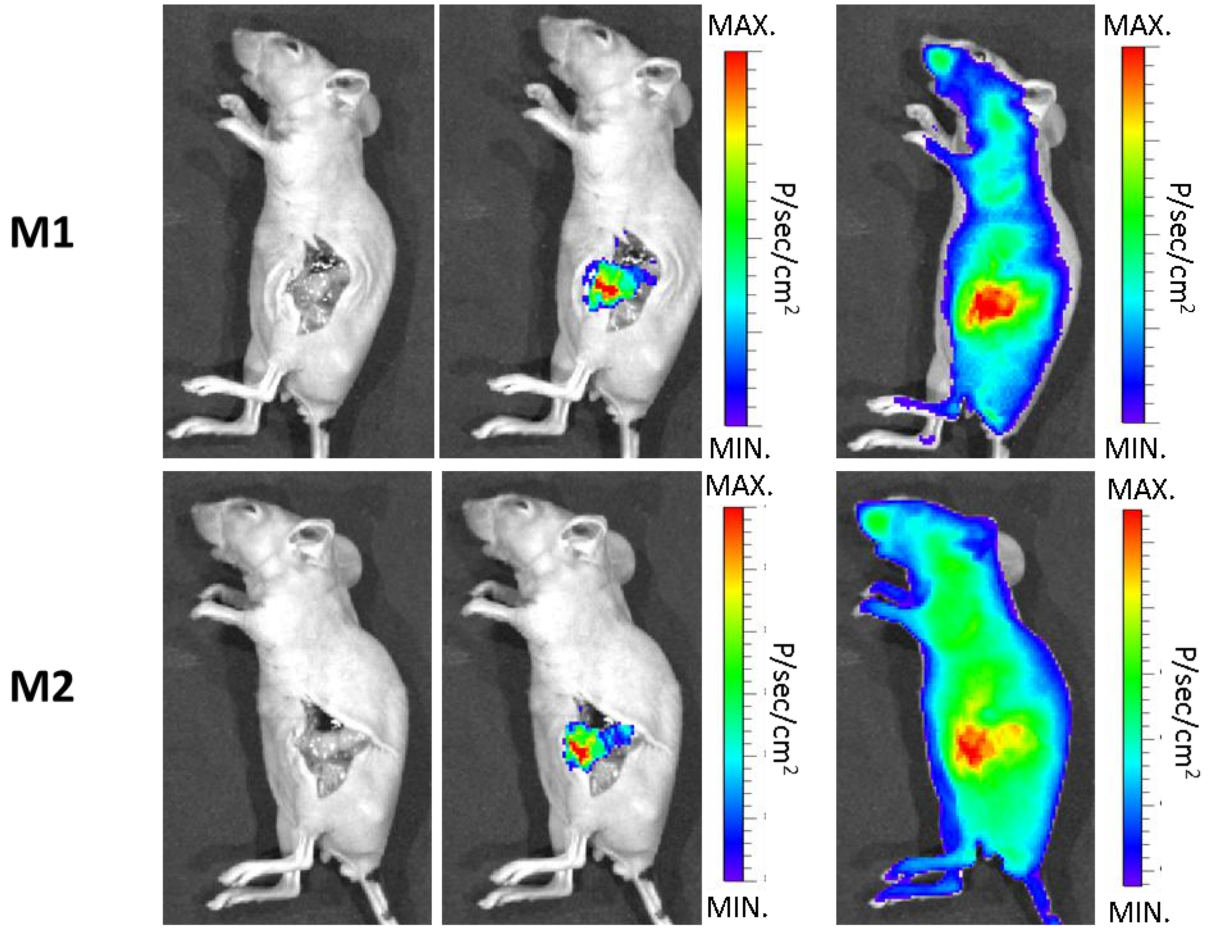


**Supplementary Fig. 23** | NIR fluorescence image of mice bearing MIA PaCa-2 cells after intravenous administration of ICG, M2 and M1 with a dose of 16 mg/kg (Fig. 7a).

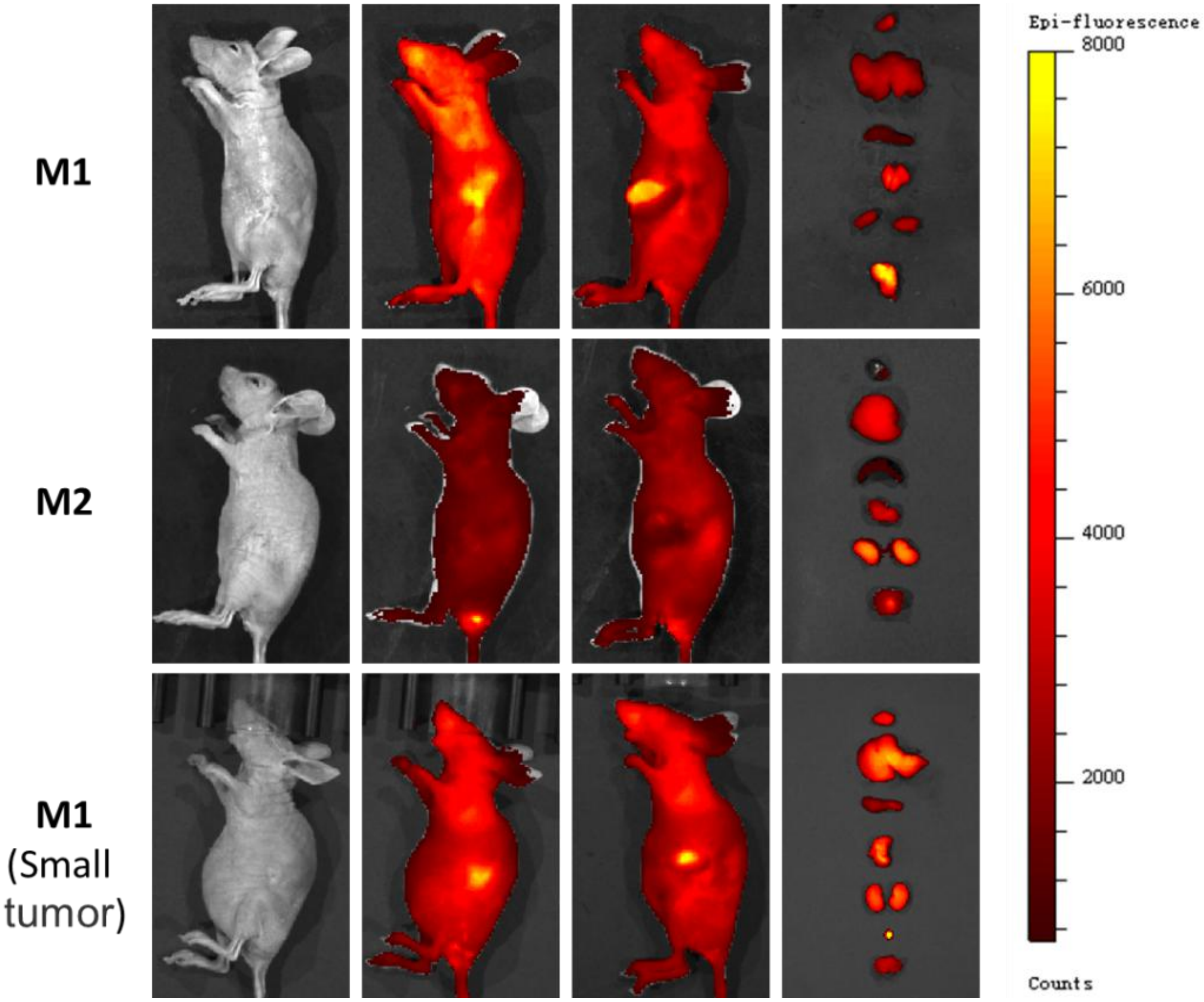


**Supplementary Fig. 24** | The *in vivo* and *in situ* NIR images of orthotopic pancreatic tumors by **M1** and **M2**, and the *ex vivo* of organ biodistribution including heart, liver, spleen, lung, kidney, and tumor after vein injection at 16mg/kg for 48 h.

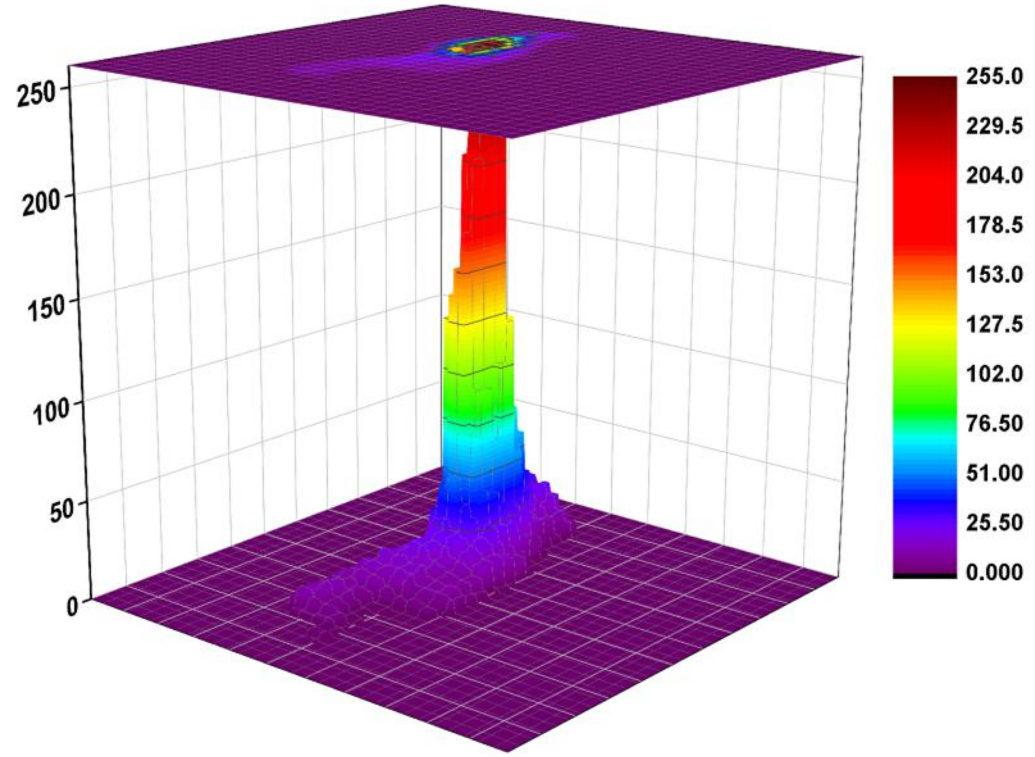


**Supplementary Fig. 25** | The 3D quantitative fluorescence intensity distribution of small size (~ 2 mm diameter) *ex vivo* orthotopic pancreatic tumor images of **M1** (Fig. 7d).

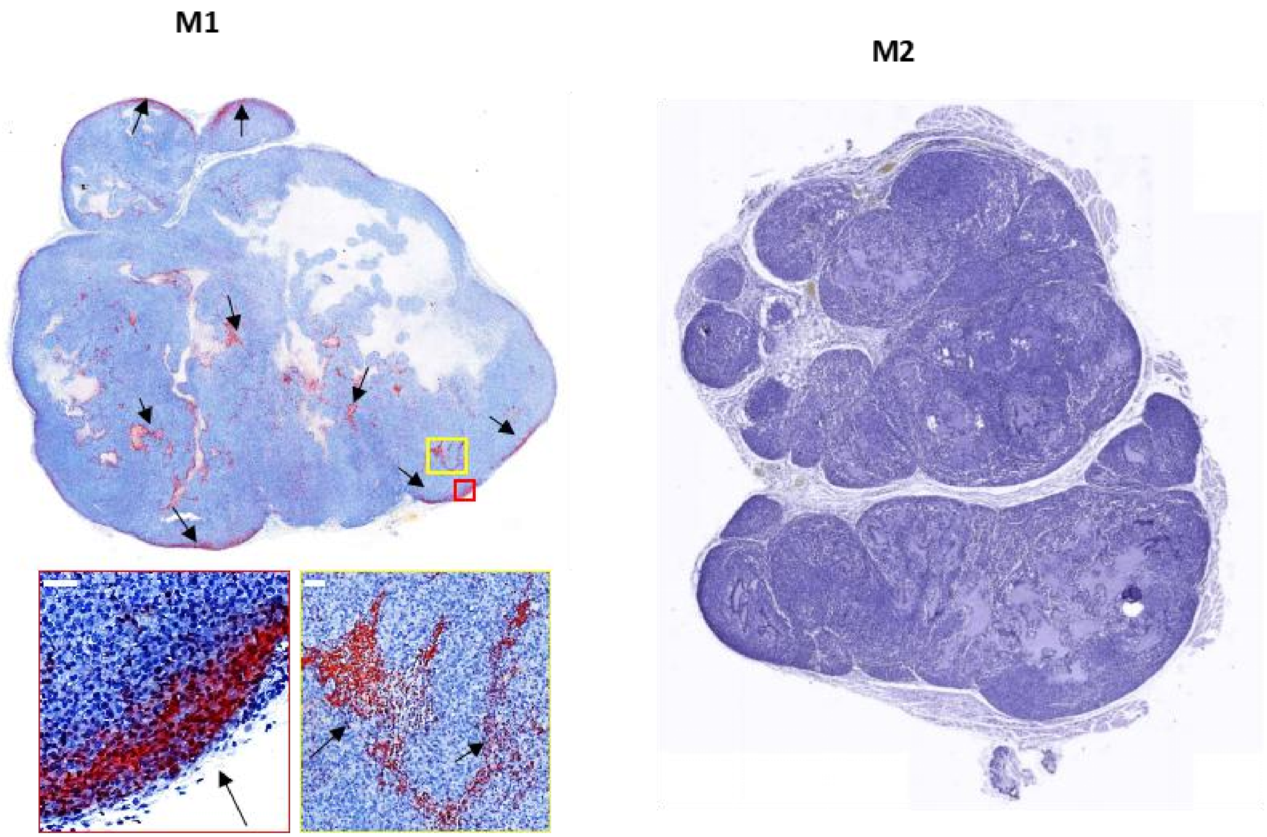


**Supplementary Fig. 26**｜ The whole tumor histologic section in mice treated with **M1** and **M2** (16 mg/kg) with congo red (red) stained assembled fibrils. Two enlarged images were respectively corresponded to the red and yellow box. Scale bar= 50 μm.

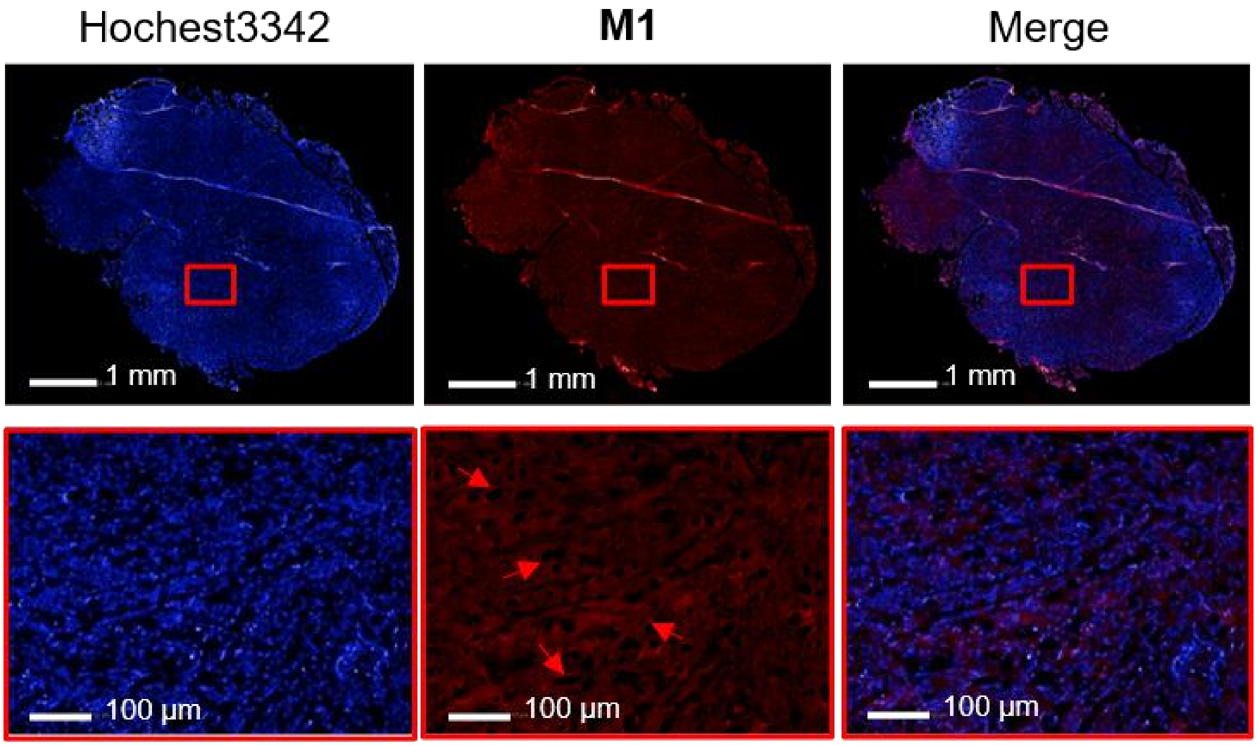


**Supplementary Fig. 27** | Fluorescence images of tumor histologic section treated with **M1** (16 mg/kg) for 48 h. Blue: Hochest 33342, Red: Cy3 labeled **M1**. Scale bar: shown in figure.

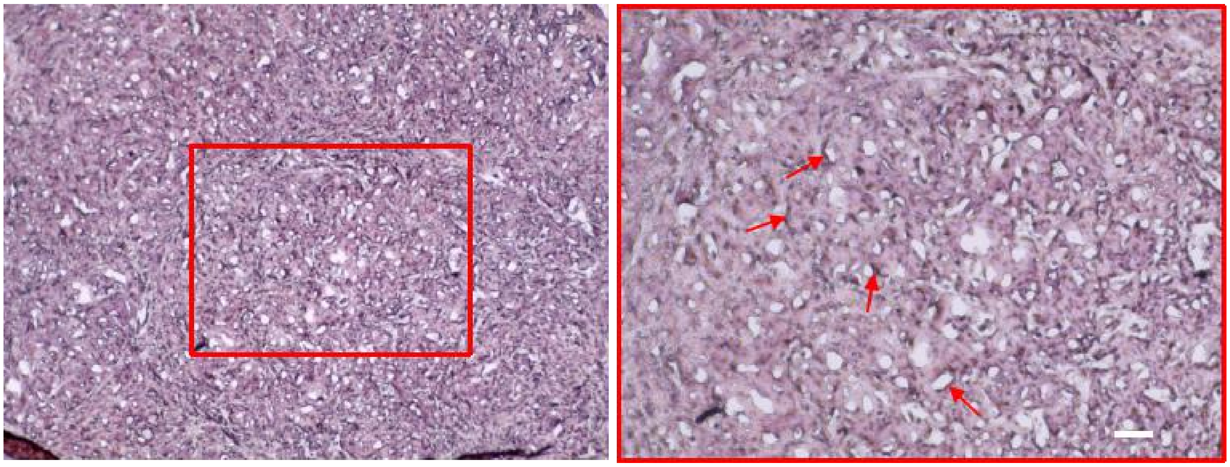

**Supplementary Fig. 28** | The tumor histologic section in mice treated with **M1** (16 mg/kg) for 48 h with congo red (red) stained assembled fibrils. A enlarged images were respectively corresponded to the red box. Scale bar: 100 μm
